# Analysis of the DNA-binding properties of Alx1, an evolutionarily conserved regulator of skeletogenesis in echinoderms

**DOI:** 10.1016/j.jbc.2021.100901

**Published:** 2021-06-19

**Authors:** Jennifer Guerrero-Santoro, Jian Ming Khor, Ayşe Haruka Açıkbaş, James B. Jaynes, Charles A. Ettensohn

**Affiliations:** 1 Department of Biological Sciences, Carnegie Mellon University, Pittsburgh, Pennsylvania, USA; 2 Department of Biochemistry and Molecular Biology, Thomas Jefferson University, Philadelphia, Pennsylvania, USA

**Keywords:** Alx1, homeodomain, protein–DNA interactions, DNA half sites, skeletogenesis, biomineralization, echinoderms, dimerization, cooperative binding, Alx1-FL, full-length Alx1, Alx1-HD, Alx1 HD alone, Alx1ΔD2, a mutant form of Alx1 that lacks the D2 domain, ChIP-seq, chromatin immunoprecipitation sequencing, CRM, *cis*-regulatory module, HD, homeodomain, His-Alx1, His-tagged Alx1, PMCs, primary mesenchyme cells, TF, transcription factor

## Abstract

Alx1, a homeodomain-containing transcription factor, is a highly conserved regulator of skeletogenesis in echinoderms. In sea urchins, Alx1 plays a central role in the differentiation of embryonic primary mesenchyme cells (PMCs) and positively regulates the transcription of most biomineralization genes expressed by these cells. The *alx1* gene arose *via* duplication and acquired a skeletogenic function distinct from its paralog (*alx4*) through the exonization of a 41–amino acid motif (the D2 domain). Alx1 and Alx4 contain glutamine-50 paired-type homeodomains, which interact preferentially with palindromic binding sites *in vitro*. Chromatin immunoprecipitation sequencing (ChIP-seq) studies have shown, however, that Alx1 binds both to palindromic and half sites *in vivo*. To address this apparent discrepancy and explore the function of the D2 domain, we used an endogenous *cis*-regulatory module associated with *Sp-mtmmpb*, a gene that encodes a PMC-specific metalloprotease, to analyze the DNA-binding properties of Alx1. We find that Alx1 forms dimeric complexes on TAAT-containing half sites by a mechanism distinct from the well-known mechanism of dimerization on palindromic sites. We used transgenic reporter assays to analyze the functional roles of half sites *in vivo* and demonstrate that two sites with partially redundant functions are essential for the PMC-specific activity of the *Sp-mtmmpb cis*-regulatory module. Finally, we show that the D2 domain influences the DNA-binding properties of Alx1 *in vitro*, suggesting that the exonization of this motif may have facilitated the acquisition of new transcriptional targets and consequently a novel developmental function.

Recent studies have highlighted the central, conserved role of Alx1, a homeodomain (HD)-containing transcription factor (TF), in the formation of the extensive, biomineralized endoskeleton characteristic of all echinoderms (1). In echinoderm clades that form skeletons at early embryonic stages, Alx1 is expressed specifically by skeletogenic cells and plays an essential role in their specification (2, 3, 4). In euechinoids, where Alx1 function has been especially well studied, the protein is a pivotal, early component of a well-characterized gene regulatory network deployed in skeletogenic primary mesenchyme cells (PMCs) (1). Alx1 provides direct, positive inputs into a large fraction (more than one-third) of all genes selectively expressed by PMCs and indirect, positive inputs into many other genes in this class, highlighting the pivotal role of Alx1 as a primary determinant of PMC identity (5, 6, 7). *Alx1* is also expressed selectively in the biomineralizing centers of adult echinoderms, pointing to a role in what is widely considered to be the ancestral mode of skeletogenesis within the phylum (8, 9, 10, 11). These observations establish *alx1* as a conserved, pivotal regulator of echinoderm skeletogenesis. Strikingly, the closest relatives of echinoderm *alx1* in vertebrates (*Cart1/alx1*, *alx3*, and *alx4*) also control the development of skeletal structures, including facial bones and the scapula, in mice (12, 13, 14, 15, 16), humans (17, 18, 19, 20, 21), and other vertebrates (22, 23). Thus, *alx1*-related genes have a conserved role in the control of biomineral formation across chordates (24).

The echinoderm *alx1* gene arose early in the evolution of the phylum *via* a gene duplication event, and Alx1 secondarily acquired the skeletogenic function that it currently exhibits in all echinoderms (11, 25). Acquisition of this new regulatory function was associated with the exonization of a 41–amino acid motif (the D2 domain) located between the HD and C terminus of Alx1 (25). The Alx1 paralog, Alx4, lacks this motif, but, remarkably, experimental insertion of the D2 domain is sufficient to confer robust skeletogenic function on Alx4. Thus, Alx1 provides a particularly striking example of an evolutionary change in TF sequence that has led to a major shift in developmental function (26). Although Khor and Ettensohn (2017) established an important role for the D2 domain *in vivo*, the biochemical function of this motif is unknown. In addition, although previous work has shown that *alx1* and *alx4* encode HD proteins with markedly different functional properties, in euechinoids, these two genes are transiently coexpressed by PMCs during gastrulation (5, 6, 11), raising the possibility that their protein products might interact physically and/or functionally.

Alx1 contains a paired-class HD of the glutamine-50 (Q50) type (27). *In vitro* binding studies have shown that paired-class HD proteins bind preferentially to palindromic sites that contain inverted TAAT sequences (28, 29). This interaction involves the cooperative binding of two protein molecules and the formation of a trimeric protein–DNA complex, requires only the HDs of the two protein molecules, and can be mediated by homodimers or heterodimers of paired-class HDs. These same studies also revealed a preference of Q50 paired-class HDs for palindromic sites in which the inverted TAAT half-sites are separated by three base pairs (P3 sites). *In vitro* binding studies with full-length human Cart1/Alx1 confirmed a preference for palindromic TAAT/ATTA sequences with 3 to 4 intervening base pairs, and reporter assays showed that such palindromic sites can mediate Cart1-based transcriptional activation (30, 31). Despite the evidence pointing to the importance of dimerization on palindromic sites, other *in vitro* binding studies have shown that paired-class HD proteins, including the vertebrate Cart1/Alx3/Alx4 proteins, are capable of interacting with half sites (also called monomeric sites), albeit with lower affinity (32). A recent, high-throughput reporter analysis of binding sites for CRX, a paired-class HD protein, found that dimeric binding sites were generally associated with stronger enhancer activity than monomeric sites and confirmed the importance of three base pair spacing in palindromic sites *in vivo* (33). The factors that regulate the binding of paired-class HD TFs to palindromic and half sites *in vivo*, and possible differences in the contributions of palindromic and half sites to enhancer activity, remain important open questions. These questions are also highly relevant to the newly evolved, skeletogenic properties of Alx1, as chromatin immunoprecipitation sequencing (ChIP-seq) analysis has shown that Alx1 binds to both palindromic and half sites *in vivo* (7).

In this study, we have examined the DNA-binding properties of Alx1, with special attention to the possible role of the D2 domain, and have compared these properties to the DNA-binding activity of the paralogous Alx4 protein. Using an endogenous *cis*-regulatory module (CRM) associated with *Sp-mtmmpb*, a gene that encodes a PMC-specific metalloprotease, and EMSA analysis, we show that Alx1 binds directly to several half sites. Moreover, Alx1 forms dimeric complexes at these sites and does so by a mechanism distinct from the well-known mechanism of dimerization that occurs at palindromic sites. We provide evidence that dimerization of Alx1 on half sites is mediated by DNA-independent, protein–protein interactions. To explore the potential functional role of half sites *in vivo*, we used transgenic reporter assays to carry out a mutational dissection of the *Sp-mtmmpb* CRM. The single palindromic site in this CRM is entirely dispensable for activity; instead, two half-sites (sites A and B) mediate PMC-specific reporter expression, and these two sites act independently and largely redundantly. Finally, we compared the DNA-binding properties of Alx1, Alx4, and a mutant form of Alx1 that lacks the D2 domain (Alx1ΔD2), using a validated, palindromic Alx1-binding site. We show that the D2 domain influences the DNA-binding behavior *in vitro* and propose that this may have supported the neo-functionalization of Alx1 by facilitating the acquisition of new transcriptional targets.

## Results

### Alx1 binds directly to endogenous half sites and can dimerize on those sites

**Figure 1:**
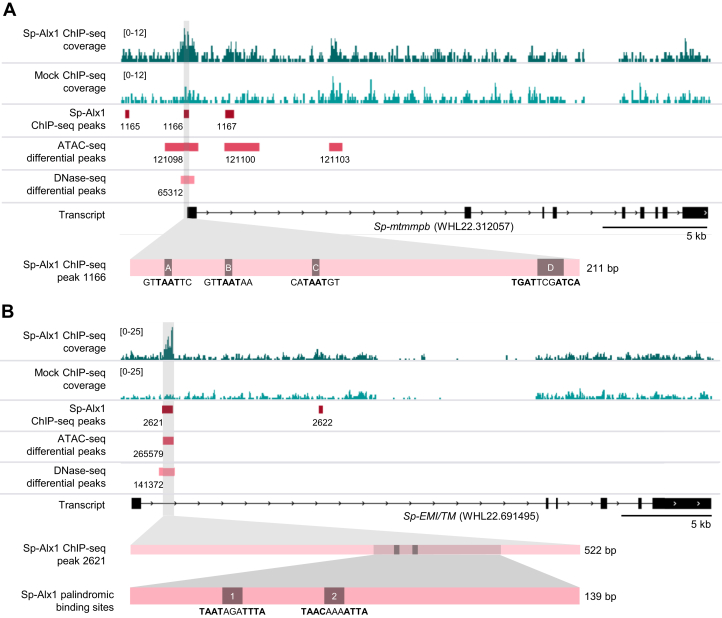
**Sp-Alx1 ChIP-seq peaks in the *Sp-mtmmpb* CRM used to identify Alx1-binding sites.***A*, genome tracks showing the location of the 210-bp Sp-Alx1 ChIP-seq peak (1166) in the 5′ untranslated region of *Sp-mtmmpb*, overlapping ATAC-seq and DNase-seq differential peaks (*i.e.*, regions of chromatin selectively accessible in skeletogenic PMCs, cells that specifically express *Sp*-*mtmmpb*). Four putative Alx1-binding sites are present within ChIP-seq peak 1166. *B*, previously identified Sp-Alx1 ChIP-seq peak within an intron of *Sp-EMI/TM*. This region drives PMC-specific reporter expression in transgenic embryos, and expression depends on the first palindromic site (7). The DNA sequence of the first site was used in the EMSA assays. CRM, *cis*-regulatory module; PMCs, primary mesenchyme cells.

One of the CRMs identified in our ChIP-seq analysis of Alx1-binding sites in *Strongylocentrotus purpuratus* (*Sp*) embryos (7) contained four putative binding sites—three half sites and one palindromic site. This 210-bp ChIP-seq peak was located near the 5′ end of *Sp-mtmmpb*, a gene that encodes a PMC-specific metalloprotease (Fig. 1*A*). The *Sp-mtmmpb* ChIP-seq peak overlapped regions of chromatin previously shown to be hyperaccessible in PMCs relative to other embryonic cell types, based on ATAC-seq and DNase-seq studies (1). Most importantly, this CRM was found to drive reporter gene expression specifically in PMCs in transgenic embryos (7).

**Figure 2:**
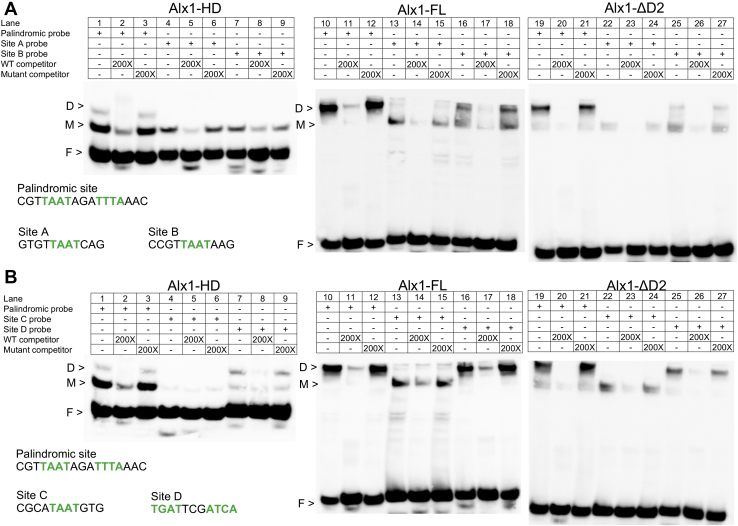
**EMSA analysis of the binding of purified Alx1-HD, Alx1-FL, and Alx1-ΔD2 proteins to the *Sp-EMI/TM* palindromic site and putative half sites.** Binding specificity was confirmed by adding WT or mutant competitor. *A*, biotin-labeled probes containing the palindromic site, site A, or site B. *B*, biotin-labeled probes containing the palindromic site, site C, or site D. For the complete sequences of all probes used in this study, see Table S1. A sample of each purified protein was separated on SDS-PAGE gels and visualized by Coomassie staining (Fig. S1). Alx1-FL, full-length Alx1; Alx1-HD, Alx1 HD alone; Alx1ΔD2, a mutant form of Alx1 that lacks the D2 domain; D, dimer; F, free probe; M, monomer.

**Figure 3:**
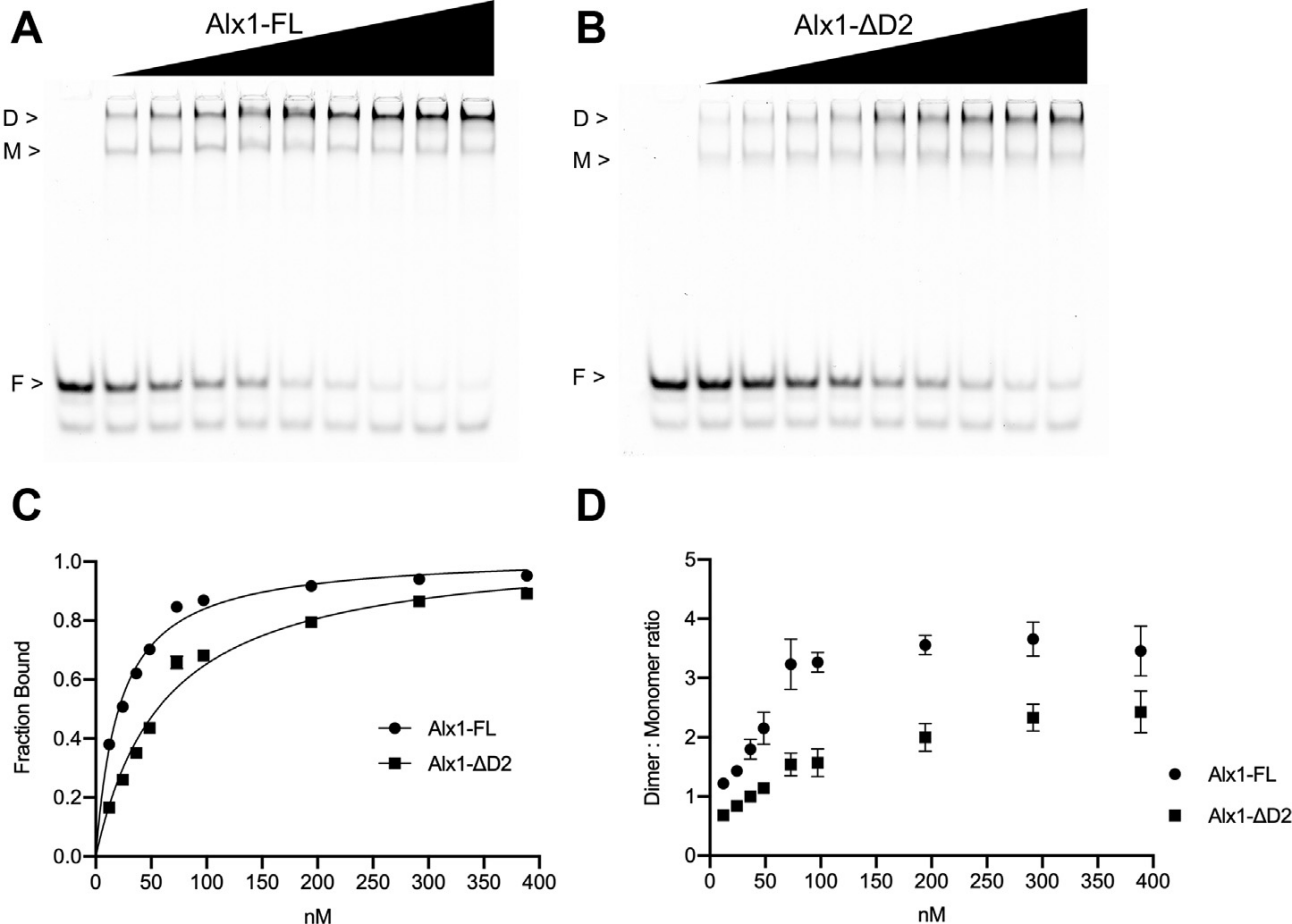
**Quantitative EMSA analysis of the binding of purified Alx1-FL and Alx1-ΔD2 proteins to the *Sp-EMI/TM* palindromic site.***A*, protein titration of Alx1-FL with a constant amount of Cy5-labeled probe containing the *Sp-EMI/TM* palindromic site. *B*, protein titration of Alx1-ΔD2 with a constant amount of Cy5-labeled probe containing the *Sp-EMI/TM* palindromic site. *C*, quantification of representative gels as shown in panels *A* and *B*. Plot of the fraction of probe bound as a function of protein concentration. Data are based on two independent replicates of the binding assay and are represented as the mean ± 1 SD. The *filled circles* denote Alx1-FL, and the *filled squares* denote Alx1-ΔD2. *D*, dimer to monomer ratios based on quantification of the same two independent replicates shown in panel *C* with data represented as the mean ± 1 SD. Alx1-FL, full-length Alx1; Alx1-HD, Alx1 HD alone; Alx1ΔD2, a mutant form of Alx1 that lacks the D2 domain; D, dimer; F, free probe; M, monomer.

**Figure 4:**
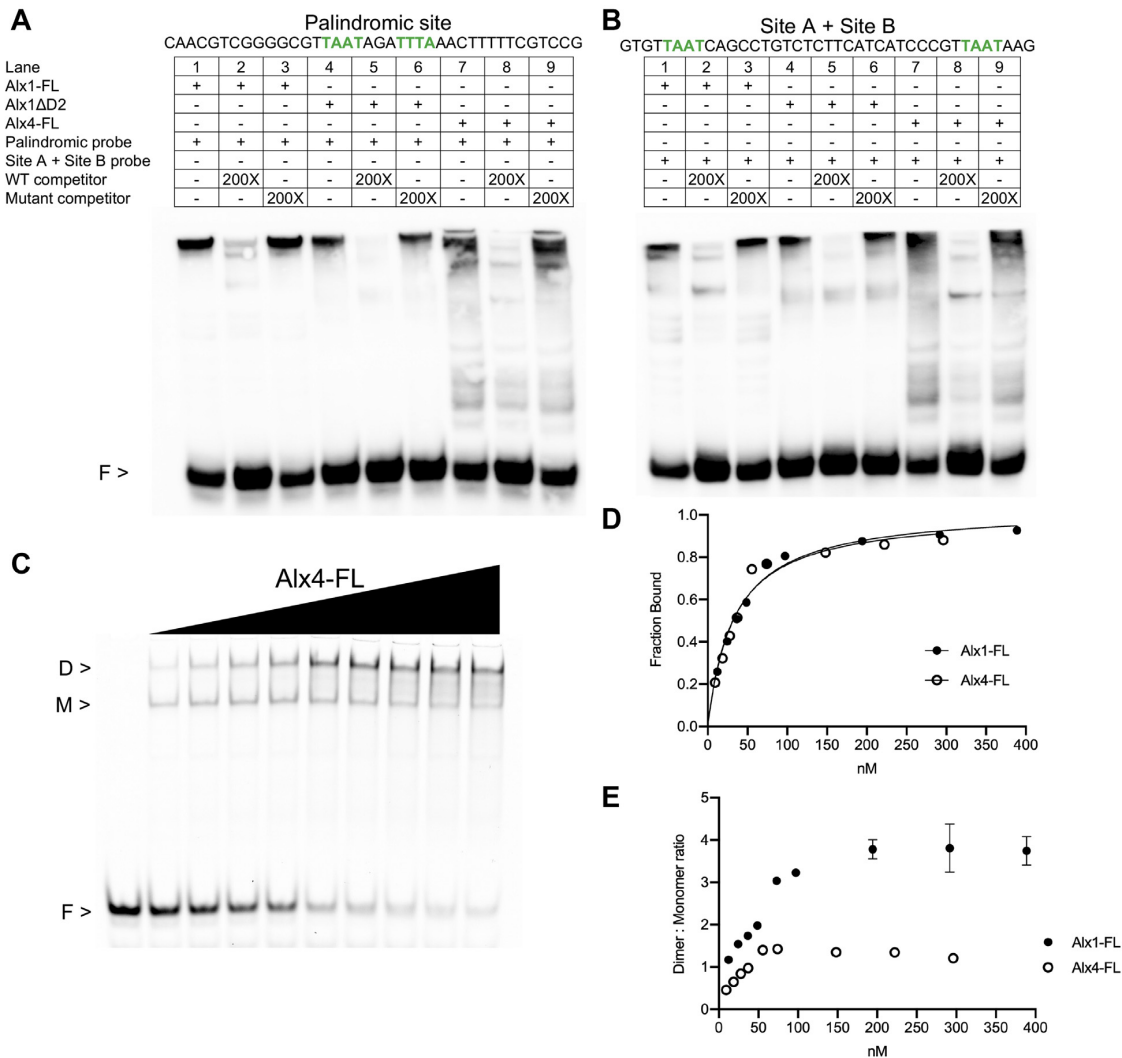
**EMSA analysis comparing the binding of purified Alx1-FL and Alx4-FL proteins to DNA.** Probes used in this analysis were all 70 bp in length and included the *Sp-EMI/TM* palindromic site or two half sites (site A and site B) from the *Sp-mtmmpb* CRM. *A* and *B*, the binding capacities of Alx1, Alx1ΔD2, and Alx4 were analyzed for each biotin-labeled DNA probe. Binding specificity was confirmed by adding WT or mutant competitor. *C*, protein titration of Alx4-FL with a constant amount of the Cy5-labeled probe containing the *Sp-EMI/TM* palindromic site. *D*, plot of the fraction bound as a function of protein concentration, based on quantification of representative gels as shown in panel *C*. Data are based on two independent replicates of the binding assay and are represented as the mean ± 1 SD. The *filled circles* denote Alx1-FL, and *empty circles* denote Alx4-FL. Data for Alx1-FL are also based on two independent replicates, distinct from those shown in Figure 3. *E*, dimer-to-monomer ratios based on quantification of the same binding assays shown in panel *C* with data represented as the mean ± 1 SD. For the complete sequences of all probes used in this study, see Table S1. Alx1-FL, full-length Alx1; CRM, *cis*-regulatory module; D, dimer; F, free probe; M, monomer.

**Figure 5:**
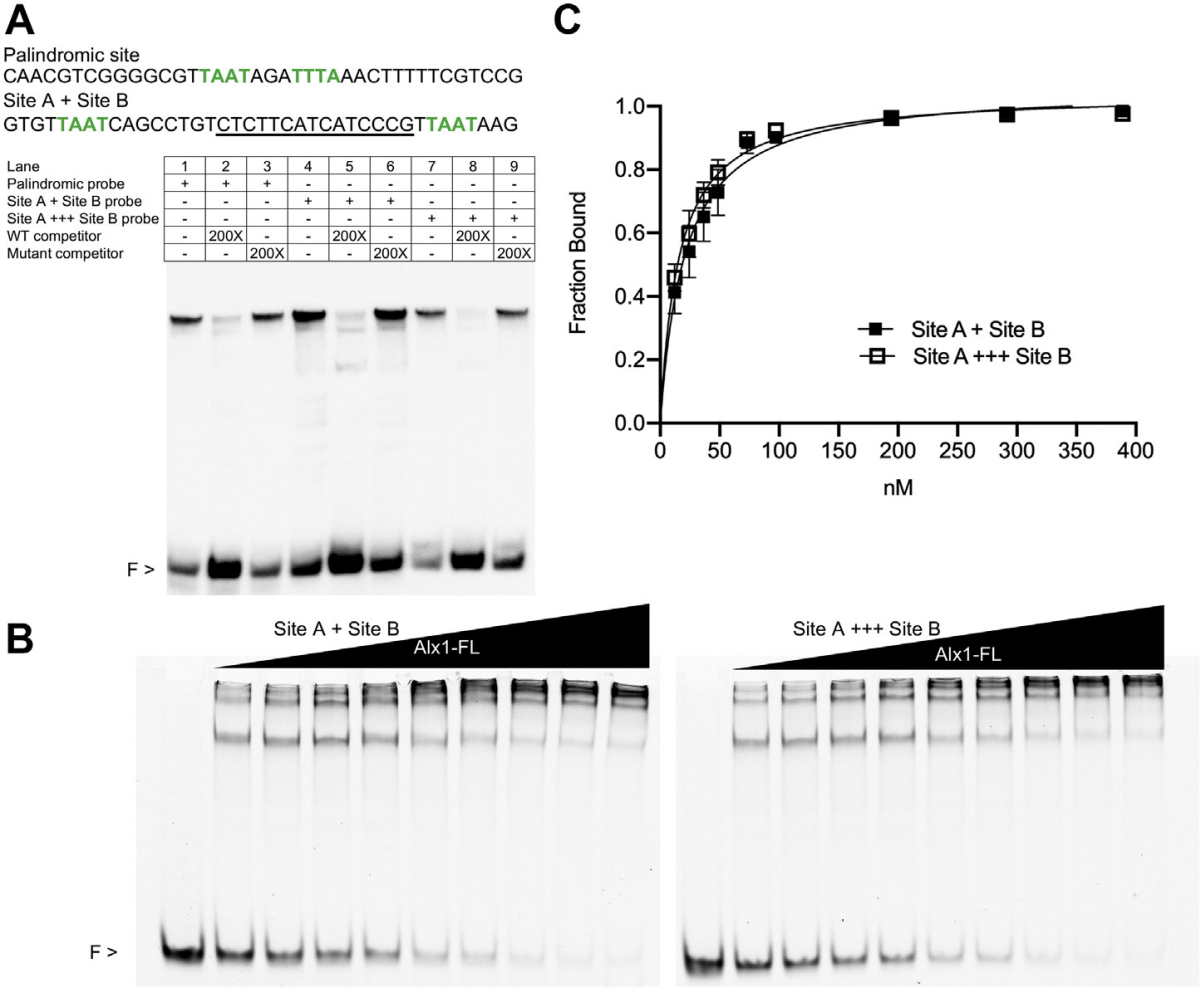
**EMSA analysis demonstrating that binding of Alx1 to closely spaced half sites is noncooperative.***A*, all probes used were 70 bp in length and included the *Sp-EMI/TM* palindromic site or two half sites (site A and site B) from the *Sp-mtmmpb* CRM. Additional nucleotides underlined were inserted between site A and site B to increase the distance between the two sites and alter their relative orientation on the DNA helix. The added sequence was designed by repeating the sequence *underlined* in the probe containing the two half sites to synthesize site A +++ site B probe. Binding specificity was confirmed by adding WT or mutant competitor. *B*, protein titration of Alx1-FL with constant amounts of two different Cy5-labeled probes containing sites A and B. *C*, quantification of the representative gel in panel *B*. Plot of the fraction bound as a function of protein concentration of two independent replicates with the data represented as the mean ± SD. The *filled squares* denote the probe site A + site B, and the *empty squares* denote the probe site A +++ site B. For the complete sequence of all probes used in this study, see Table S1. Alx1-FL, full-length Alx1; CRM, *cis*-regulatory module; F, free probe.

We examined the binding of Alx1 to each of the four putative Alx1-binding sites in the *Sp-mtmmpb* CRM using an EMSA. For each probe tested, binding specificity was assessed by adding excess nonbiotinylated, competitor DNA that had the same sequence as the labeled probe or that had mutations introduced into the putative Alx1-binding site. The binding of full-length Alx1 (Alx1-FL), the Alx1 HD alone (Alx1-HD), and Alx1ΔD2 was assayed. As an additional control, we used an endogenous, palindromic Alx1-binding site located within a CRM associated with *Sp-EMI/TM*, a gene that encodes a novel, PMC-specific transmembrane protein (Fig. 1*B*). This Alx1-binding site was previously shown to be required for the activity of a transcriptional reporter in transgenic sea urchin embryos (7). It should be noted that although this sequence is not perfectly palindromic, the single nucleotide difference does not prevent dimerization of Alx1 (Figure 2, Figure 3, Figure 4, Figure 5), consistent with previous studies, which showed that slight deviations from palindromic sequences can be accommodated without substantially affecting cooperative binding and dimer formation, provided spacing between the half sites is preserved (28, 29).

Alx1-HD bound to the *Sp-EMI/TM* palindromic site (Fig. 2, *A* and *B*, lanes 1–3) and site D (Fig. 2*B*, lanes 7–9) in both monomeric and dimeric protein–DNA complexes. This observation was consistent with what has been reported for other paired-class HDs, which can bind cooperatively to palindromic sites (28, 29, 34). Based on these findings, we concluded that site D, which has the sequence **TGAT**TCG**ATCA**, functions as a canonical palindromic site. Alx1-HD also bound to sites A and B but only as a monomer (Fig. 2*A*, lanes 4 and 7, respectively), and this protein fragment interacted very weakly, if at all, with site C (Fig. 2*B*, lane 4).

When Alx1-FL and Alx1-ΔD2 were analyzed by EMSA, the predominant molecular complexes that formed on the *Sp-EMI/TM* palindromic site were dimeric protein–DNA complexes (Fig. 2*A*, lanes 10 and 19; Fig. 2*B*, lanes 10 and 19). This was also true in the case of site D (Fig. 2*B*, lanes 16 and 25), further supporting the conclusion that site D functions as a palindromic site. Monomeric protein–DNA complexes were detected on sites A, B, and C (Fig. 2*A*, lanes 13, 16, 22, and 25; Fig. 2*B*, lanes 13 and 22). Significantly, a slower migrating complex also formed in association with each of these sites and was especially prominent in the case of site B (Fig. 2*A*, lanes 16 and 25). This larger complex exhibited a mobility indistinguishable from that of the protein dimer bound to a palindromic site. We therefore attributed the formation of this complex to protein–protein interactions that promoted Alx1 dimerization on half sites, although presumably only one Alx1 molecule was bound tightly to DNA at these sites *via* its HD. We also noted that, although Alx1-HD did not exhibit appreciable binding to site C, Alx1-FL (and Alx1-ΔD2) interacted with this site to form primarily a monomeric complex (Fig. 2*B*, lanes 13 and 22). This suggests that binding of Alx1 to site C is influenced by domains of the protein other than the HD.

In initial studies, we found that deletion of the D2 domain did not prevent the formation of monomeric and dimeric complexes on the *Sp-EMI/TM* palindromic site and on the four Alx1-binding sites in the *Sp-mtmmpb* CRM (Fig. 2*A*, lanes 10–27; Fig. 2*B*, lanes 10–27). In several cases, however, it appeared that the amounts of the complexes and/or the ratio of monomeric to dimeric complexes was affected by deletion of the D2 domain. To examine this issue further, we used quantitative EMSAs to compare the ability of Alx1-FL and Alx1-ΔD2 to bind to a Cy5-labeled *Sp-EMI/TM* palindromic site (Fig. 3). We measured the active fraction of protein in each preparation (see Experimental procedures) and titrated each protein over a wide concentration range. These studies showed that the deletion of the D2 domain had a reproducible effect on the binding behavior of Alx1 (Fig. 3, *A*–*C*) and significantly reduced the ratio of dimeric complex to monomeric complex at all protein concentrations tested (Fig. 3*D*).

### Alx1–Alx1 interactions in the absence of DNA

**Figure 6:**
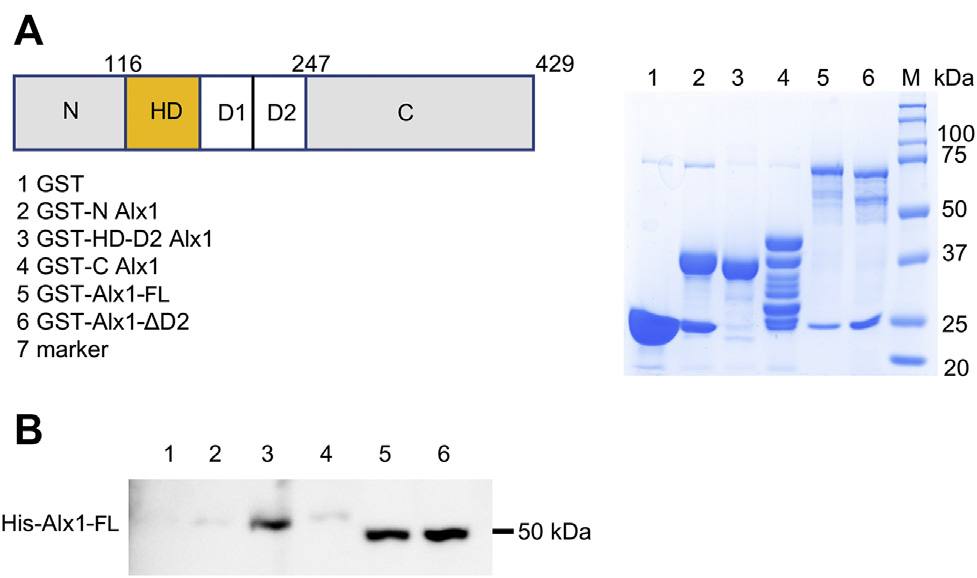
**Alx1–Alx1 interactions in the absence of DNA.***A*, GST-tagged Alx1 protein and various deletions mutants were expressed in *Escherichia coli* and immobilized on glutathione beads. The protein samples were separated on SDS-PAGE gels and visualized by Coomassie staining. *B*, GST-tagged, immobilized proteins shown in panel *A* were incubated with purified full-length, His-tagged Alx1. The beads were washed, separated on an SDS-PAGE gel, and analyzed by Western blotting using an anti-His antibody.

EMSAs indicated that Alx1 formed dimers on half site B (and, to a lesser extent, on half sites A and C) by a mechanism distinct from the HD-mediated dimerization that occurs on palindromic sites. It seemed plausible that dimerization on a half-site might involve direct (DNA independent) interactions between two Alx1 monomers. Previous studies on Cart1, a vertebrate ortholog of Alx1, indicated that this protein can dimerize in the absence of DNA (35). We therefore performed glutathione-*S*-transferase (GST)-pulldown experiments using Alx1-FL and four shortened forms of the protein: Alx1-Nterm (which consisted of the 116 amino acids N-terminal to the HD), Alx1-HD-D2 (which consisted of only the HD, D1, and D2 domains), Alx1-Cterm (which consisted of the 183 amino acids C-terminal to the D2 domain), and Alx1ΔD2. A pulldown using GST alone was used as a negative control. GST-tagged proteins (or GST) were first immobilized on glutathione beads and then incubated with purified, His-tagged, Alx1-FL (His-Alx1). We found that all GST-tagged Alx1 protein fragments were subject to varying degrees of degradation (Fig. 6*A*). These experiments showed that the N- and C-terminal regions of Alx1 did not bind at detectable levels to full-length, His-tagged Alx1, but the full-length protein bound (Fig. 6*B*, lanes 2, 4, and 5, respectively). The central region consisting of only the HD, D1, and D2 domains was also capable of binding (Fig. 6*B*, lane 3). Deletion of the D2 domain alone did not prevent binding to the full-length protein (Fig. 6*B*, compare lanes 5 and 6), and binding was also detected between Alx1-ΔD2 and Alx1-ΔD2 (Fig. S2), demonstrating that the D2 domain of Alx1 was not required for DNA-independent interactions.

### Alx1 and Alx4 have similar but distinct DNA-binding properties and can form heterodimers

Given that Alx1 and Alx4 have distinct developmental functions, we compared the DNA-binding properties of the two proteins by EMSA. We found that Alx4 bound to the *Sp-EMI/TM* palindromic site as a dimer (Fig. 4*A*, lane 7), as did Alx1-FL and Alx1-ΔD2 (Fig. 4*A* lanes 1 and 4, respectively). In the case of Alx4, a slightly faster migrating complex was detected because of the lower molecular weight of this protein. Moreover, Alx4 was able to bind a DNA probe that contained half sites A and B (Fig. 4*B*, lane 7), as did Alx1-FL and Alx1-ΔD2 (Fig. 4*B*, lanes 1 and 4). Although the binding behaviors of Alx1-FL and Alx4-FL appeared qualitatively similar, quantitative analysis of the binding of the two proteins to a Cy5-labeled palindromic probe showed that Alx4-FL exhibited a markedly lower dimer-to-monomer ratio across a wide range of protein concentrations (Fig. 4, *C*–*E*).

GST-pulldown experiments showed that Alx1 and Alx4 were capable of interacting directly in the absence of DNA (Fig. S3). We next tested whether these two proteins could form heterodimers on DNA, using the *Sp-EMI/TM* palindromic Alx1 site as a target. When mixed, Alx1 and Alx4 formed heterodimers that migrated in gels at a position between Alx1:Alx1 and Alx4:Alx4 homodimers (Fig. S4*A*). The presence of Alx1:Alx4 heterodimers was confirmed by antibody super-shift experiments (Fig. S4*C*). These studies showed that, at least *in vitro*, Alx1 and Alx4 are capable of forming heterodimers on DNA. As discussed below, however, the normal developmental expression patterns of *alx1* and *alx4* strongly suggest that, *in vivo*, the homodimeric form of Alx1 predominates over the heterodimeric form during PMC specification (see Discussion).

### Binding of Alx1 to closely spaced half sites is noncooperative

As noted above, studies on paired-class HD proteins have demonstrated that binding to palindromic sites occurs in a cooperative fashion. We noted that half sites A and B were separated by only 24 bp (~80 Å) in genomic DNA, raising the possibility that two Alx1 molecules could interact directly with one another across this distance (36) and bind cooperatively. Using EMSA, we explored the possibility that Alx1 bound cooperatively to a probe containing site A and site B by examining the effect of increasing the spacing between these two sites. An additional 15 bp were introduced between the two half sites by inserting a direct repeat of part of the endogenous sequence that separates these sites. This insertion not only increased the distance separating the two half sites by >60% but altered the relative position of the sites on the DNA molecule by half of a helical turn. As a positive control, we used a DNA probe of equal length that contained the *Sp-EMI/TM* palindromic Alx1-binding site, flanked by endogenous genomic sequence. We observed the formation of dimeric protein–DNA complexes with all three DNA probes tested (Fig. 5*A*, lanes 1, 4, and 7). Quantitative EMSA analysis confirmed that altering the spacing and relative orientation of sites A and B had very little or no effect on Alx1 binding (Fig. 5, *B* and *C*).

We interpreted the pattern of complex formation on the combined A and B sites as the binding of one Alx1 molecule to each half site, which would be expected to create a protein–DNA complex with the same electrophoretic mobility as a complex containing two Alx1 molecules bound to a single palindromic site. Because substantial dimer formation was observed on site B alone (Fig. 2*A*, lane 16), however, we also predicted that higher order complexes might form on a probe containing site A and site B. To visualize such complexes, we lowered the overall mass by using shorter probes (55 bp instead of the 70 bp used in Fig. 5). Under these conditions, we detected complexes that migrated more slowly than Alx1 dimers bound to a palindromic site (Fig. S5, lanes 1 and 4).

### Transgenic reporter assays reveal a function for Alx1 half sites *in vivo*

**Figure 7:**
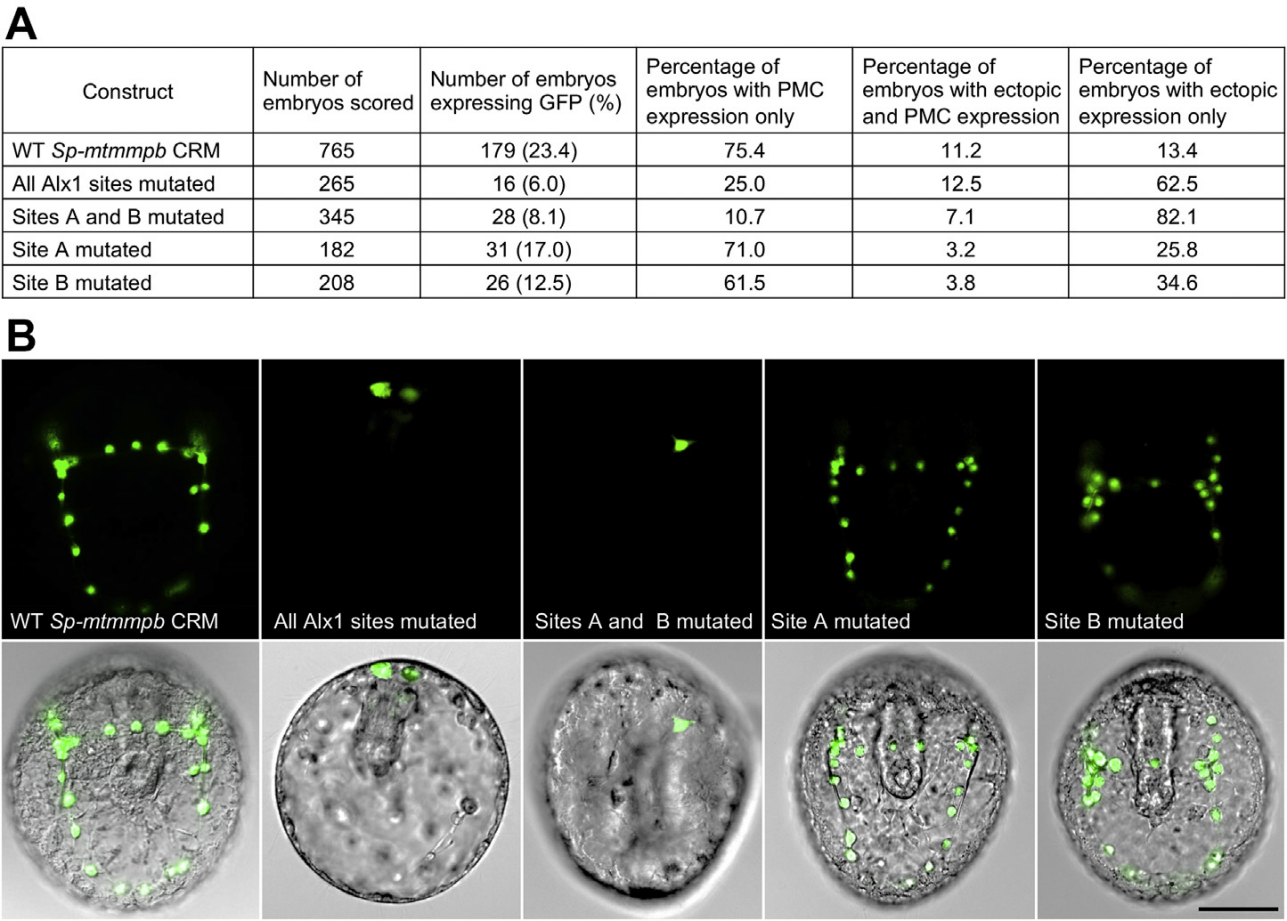
**Analysis of the functional role of half sites *in vivo* using transgenic reporter assays.***A*, GFP expression in embryos injected with WT *Sp-mtmmpb* CRM and different Alx1-binding site mutants assayed by fluorescence microscopy. *B*, representative embryos showing GFP expression. The scale bar represents 50 μm. CRM, *cis*-regulatory module; PMC, primary mesenchyme cell.

We examined the *in vivo* functions of the various Alx1-binding sites in the *Sp-mtmmpb* CRM using transgenic reporter assays (Fig. 7). We injected fertilized *Sp* eggs with linearized EpGFPII plasmids that contained the endogenous 210 bp CRM (or mutant forms of the CRM) cloned upstream of a basal promoter and the coding sequence of GFP. Embryos were collected 48 h after fertilization (prism stage) and examined by fluorescence microscopy. Foreign DNA is incorporated and expressed in a highly mosaic fashion in sea urchin embryos; this requires the analysis of populations of embryos to reconstruct the expression patterns of transgenes (37). As reported previously, the endogenous *Sp-mtmmpb* CRM drove robust expression of GFP, which was almost entirely restricted to PMCs (~75% of GFP-expressing embryos; see Fig. 7*A*). In contrast, a form of the CRM that had all four Alx1-binding sites mutated failed to support appreciable GFP expression. Notably, injection of a form of the CRM that contained mutated A and B sites (but with intact C and D sites) also resulted in dramatically reduced numbers of GFP-expressing embryos, and reporter expression was no longer restricted to PMCs. Mutation of the A and B half sites individually resulted in reduced numbers of GFP-expressing embryos and a reduced fraction of these embryos exhibited PMC-restricted expression, but both constructs drove PMC-specific expression in a substantial fraction of embryos (~60–70% of GFP-expressing embryos). These findings show that Alx1 half sites play an important role *in vivo* in controlling the lineage-restricted activity of the *Sp-mtmmpb* CRM and that sites A and B have partially redundant functions in driving the activity of this module in PMCs.

## Discussion

### Paired-class HD TF usage of palindromic and half sites

The cooperative binding of paired-class HD proteins to palindromic DNA sites with the consensus sequence TAATNNNATTA (also known as a P3 site) has been well described (28, 29, 34). *In vitro* studies using purified Cart1 and Alx4, members of the Alx1 gene family, demonstrate a preferred interaction with this palindromic sequence (30, 31). Our ChIP-seq studies, however, indicate that the majority of Alx1-binding sites *in vivo* do not match this consensus sequence and instead contain putative half sites with the consensus core sequence TAAT (7). Indeed, despite much evidence pointing to the importance of palindromic sites, other *in vitro* binding studies have indicated that paired-class HD proteins, including members of the Alx1 family, are capable of interacting with half sites (28, 32). Notably, Perez-Villamil *et al.* (38) showed that Alx3 binds to half sites associated with the somatostatin promoter and that these sites can modulate the transcriptional activity of reporter constructs in cell lines. More recently, high-throughput reporter studies of Crx-binding sites demonstrated binding to both half sites and palindromic sites, although palindromic sites were associated with stronger enhancer activity (33). The factors that operate *in vivo* to regulate the binding of paired-class HD TFs to palindromic and half sites, and the relative contributions of these two types of binding sites to CRM activity, remain open questions.

In the present study, we analyzed a CRM associated with *Sp-mtmmpb*, a gene expressed specifically in PMCs that receives essential, positive inputs from Alx1 (6). ChIP-seq analysis shows that Alx1 binds directly to the 210 bp CRM *in vivo*, and this DNA fragment is sufficient to drive robust expression of GFP selectively in PMCs in transgenic embryos (7). We found that the *Sp-mtmmpb* CRM contains a single, perfectly palindromic site which, although it diverges from the canonical TAATNNNATTA sequence, supports HD dimerization *in vitro* and therefore presumably functions as a typical P3 site. Surprisingly, we found that this site was not required for robust, PMC-specific expression in transgenic embryos. In contrast, deletion of both Alx1 half sites (sites A and B) in the same construct dramatically reduced reporter expression. Our studies demonstrate the functional significance of Alx1 half sites *in vivo* and show that, at least in the context of the 210 bp *Sp-mtmmpb* CRM, such sites make a greater overall contribution to regulatory control than a *bona fide* palindromic site. Thus, although half sites represent relatively low-affinity binding sites *in vitro* compared to P3 sequences (30), they can play a major role in defining target specificity *in vivo* (39, 40).

How do half sites function? Although sites A and B are located close to one another, several findings argue against the hypothesis that these sites function in concert to support cooperative dimerization of Alx1 molecules by the mechanism that has been widely accepted for palindromic sites. Quantitative analysis of gel shifts showed no detectable effect of inserting additional sequence that both increased the spacing between the two sites and altered their orientation relative to one another on the DNA duplex. It is difficult to envision a mechanism by which binding of the first Alx1 molecule might induce a change in complex conformation that would bring the second site into close proximity regardless of the intervening sequence and the orientation of the two half sites on the double-helical DNA. Indeed, studies with other paired-family HD proteins have shown that the addition of even two additional base pairs between two half sites (*i.e.*, converting a P3 site into a P5 site) reduces cooperative binding (28). Moreover, we find that the pattern of complex formation on a DNA molecule with two half sites is very different from that seen on a palindromic site (Fig. S5). Additional high-mobility complexes are visible in the former case that likely represent a mixture of independent, monomeric, and dimeric binding events at each half site. This difference in complex formation also argues against the view that the two half sites are functioning like a single, palindromic site. Finally, our *in vivo* assays showed that deletion of either half-site individually had little effect on reporter gene expression, a result that would not be expected if activity were dependent upon a cooperative interaction between the two half sites. Based on all these considerations, we conclude that sites A and B function independently and redundantly.

Our EMSA analysis provides the first reported evidence of HD protein dimerization on half sites and shows that the degree of dimerization *in vitro* varies among such sites. These findings therefore blur the distinction between half sites (which are often referred to as “monomeric” sites) and palindromic (“dimeric”) sites and suggest a continuum of binding activities associated with a TAAT core sequence which can promote varying degrees of protein dimerization. Our findings indicate that this dimerization involves domains of the Alx1 protein outside the HD, as the isolated HD failed to dimerize on the same half sites. We therefore propose that direct protein–protein interactions drive dimerization on half sites, consistent with our GST-pulldown data and with other evidence that Alx1 family proteins can dimerize in a DNA-independent fashion (35). A variety of factors might influence the extent of dimer formation on half sites *in vivo*, including the local concentration of HD-containing TFs, the specific DNA sequences that flank the TAAT core, and the presence of accessory proteins. There is abundant evidence that dimerization is important for the robust, cooperative activation of palindromic, P3 sites by paired-class HD proteins, including Cart1 and Alx3 (28, 35, 38), and the formation of dimers on half sites may have a similar impact on promoter activation, although this has not been tested directly. Our analysis of the *Sp-mtmmpb* CRM did not reveal a clear correlation between the ability of Alx1-binding sites (whether palindromic or half sites) to mediate protein dimerization *in vitro* and the regulatory contributions of these sites in transgenic embryos, but the extent of dimer formation on these sites *in vivo* may be quite different because of the presence of accessory proteins.

### Evolution of Alx1 and the role of the D2 domain

Alx1 and Alx4 are paralogs that arose early in echinoderm evolution through gene duplication (11, 24, 25). The two proteins have almost identical HDs (97% amino acid identity) and share a highly conserved *otp/aristaless/rax* domain but are otherwise dissimilar in sequence. The *alx1* and *alx4* genes are coexpressed selectively by PMCs early in embryogenesis, but the onset of *alx1* expression precedes that of *alx4* by several hours, and at the time of PMC differentiation, the level of *alx1* mRNA in PMCs is >20-fold higher than that of *alx4* mRNA (5, 6). Thus, no Alx1:Alx4 heterodimers could exist early in development (*i.e.*, before the onset of *alx4* expression) and even at later stages; when our findings suggest that heterodimers could form, it seems very likely that the homodimeric form of Alx1 is more abundant.

Experimental manipulations show that even when Alx1 and Alx4 proteins are expressed at similar levels, only Alx1 has the ability to activate downstream circuitry of the skeletogenic network (25). This capacity is attributable to the D2 domain, which is ordinarily absent from the paralogous Alx4 protein but is sufficient to confer skeletogenic function on a chimeric form of Alx4 that contains the domain. The relatively recent evolutionary acquisition of D2 and the importance of this domain in endowing Alx1 with functional properties distinct from those of Alx4 originally suggested a simple hypothesis that Alx1 acquired new gene targets through D2-mediated changes in direct DNA-binding specificity. In support of this hypothesis, our studies reveal a role for the D2 domain in regulating the DNA-binding properties of Alx1, as assessed by quantitative gel-shift assays using a palindromic site shown previously to be functional *in vivo*. Significantly, we detected a decrease in the dimer-to-monomer ratio when the D2 domain was deleted from Alx1. Furthermore, full-length Alx4, which lacks a D2 domain, also exhibited a reduced ability to form dimers when compared with Alx1-FL. These findings suggest that one effect of the D2 domain is to facilitate the cooperative binding of an Alx1 molecule to an Alx1–DNA complex. Our findings do not exclude the possibility, however, that the D2 domain also has an effect on the ability of Alx1 molecules to form monomeric complexes on DNA. Although binding curves for Alx1 and Alx4 were very similar, suggesting that there was little difference in the *K*_*d*_ of monomer formation between these two proteins, they were somewhat different between Alx1-FL and Alx1ΔD2. Regardless of whether one or both mechanisms are at work, our findings clearly show that the *in vitro* DNA binding properties of Alx1 are affected by the D2 domain.

Like other members of the protein family, Alx1 can engage in DNA-independent dimerization. We did not detect a role for the D2 domain in mediating such dimerization, although GST-pulldown assays may not have detected subtle effects. In addition, our *in vitro* studies involving purified Alx-family proteins do not address the possibility that the D2 domain influences heterodimer formation with other, as-yet unidentified, protein partners. There are several examples of the regulation of HD protein target specificity through interactions with protein partners (41, 42). To date, there has been no systematic search for Alx1-interacting proteins, but vertebrate members of the Alx family can interact directly with P300/CBP (43) and a basic helix-loop-helix protein, E47 (44). Further studies will be required to determine whether Alx1 interacts with other proteins and, if so, whether the D2 domain influences such interactions. Although the findings reported here do not exclude the possibility that the D2 domain plays diverse roles *in vivo*, they provide the first evidence that this critically important, newly evolved domain has a direct role in regulating the DNA-binding behavior of Alx1. We hypothesize that the evolutionary recruitment of the D2 domain facilitated the acquisition of new transcriptional targets at least in part by modulating the DNA-binding properties of Alx1, thereby allowing the protein to adopt a novel developmental function.

## Experimental procedures

### Animals

Adult *S. purpuratus* were acquired from Patrick Leahy (California Institute of Technology). Release of gametes and culture of embryos was performed as previously described (7). Animal studies were carried out in accordance with the Carnegie Mellon University Institutional Animal Care and Use Committee.

### Plasmids

A cDNA for *Lytechinus variegatus* Alx1 was used for PCR-based amplification of segments that encoded Alx1-FL, the N-terminal region, HD, HD-D1-D2, and the C-terminal region. *L. variegatus* Alx4 and Alx1ΔD2 cDNAs were amplified by PCR from plasmids previously characterized (25). PCR also introduced restriction sites for cloning into appropriate vectors and N-terminal tags (His and Flag) for bacterial expression and purification: pTXB1 vector (Cat. No. N6707S; New England Biolabs), pET-Duet-1 (Cat. No. 71146; Novagen), and pGEX4T-1 (Cat. No. 28-9545-49; GE).

### Protein expression and purification

Plasmids were used to transform Rosetta 2 cells (Cat. No. 71400; Novagen). Bacterial cells were cultured at 37 °C and induced at an absorbance of 595 nm of 0.600 with 0.5 mM IPTG. The temperature was lowered to 16 °C, and cells were allowed to grow for an additional 3 h and then collected and centrifuged. All bacterial pellets were dissolved and sonicated in a buffer containing 300 mM NaCl, 50 mM Tris, pH 6.8, 0.5% Triton X-100, 20 mM sarcosine, 2 mM 2-mercaptoethanol, and protease inhibitor cocktail. The mixture was sonicated and then cleared through centrifugation. The lysate was diluted to lower the concentration of detergents to a final concentration of 3.3 mM sarcosine and 0.16% Triton X-100.

For the purification of Flag-Alx1-intein and Flag-Alx4-intein, the bacterial lysate was loaded into a prepacked column of pre-equilibrated Flag-M2 agarose beads (Cat. No. H0537; Sigma). Protein-bound Flag-M2 agarose beads were then washed with a buffer containing 300 mM NaCl, 50 mM Tris, pH 6.8, 0.1% Triton X-100, 2 mM 2-mercaptoethanol, and protease inhibitor cocktail. The protein was eluted with the same buffer containing Flag peptide (100 μg/ml). The eluted protein was then incubated for 2 h with pre-equilibrated chitin beads (Cat. No. S6651S; New England Biolabs) on a rocker at 4 °C. The mixture was loaded onto a column, and the beads were washed with the buffer containing 300 mM NaCl, 50 mM Tris, pH 6.8, 0.1% Tris, pH 6.8, 0.1% Triton X-100, 2 mM 2-mercaptoethanol, and protease inhibitor cocktail. To elute the protein, the column was sealed and incubated in the wash buffer with 100 mM 2-mercaptoethanol for 40 h at 4 °C. The single tagged Flag-Alx1 and Flag-Alx4 protein were then eluted, concentrated, and desalted.

Purification of His-Alx1ΔD2-intein was performed as described previously for His-Alx1-intein (7). Single-tagged His-Alx1-HD was purified in the same way, except that after the elution of the protein from the nickel beads, the sample was desalted.

Lysates of GST-tagged proteins were incubated with glutathione-agarose beads (Cat. No. 16100; Pierce) at room temperature (RT) for 1 h on a rocker. The beads were then washed several times, and the protein was left immobilized on the beads.

The concentration of each protein was determined by running the protein samples in parallel with BSA protein standards on a TGX Stain-Free gel (Bio-Rad) and using Bio-Rad Image Lab software to image the gel and quantify the bands.

### Protein–protein interactions

Immobilized GST-tagged protein were incubated overnight with His-Alx1 in a buffer containing 200 mM NaCl, 50 mM Tris, pH 6.8, 0.1% Triton X-100, 2 mM 2-mercaptoethanol, and protease inhibitor cocktail and placed on a rotating rocker at 4 °C. The beads were washed several times and resuspended in the loading buffer, and samples were separated on 10% polyacrylamide gels. Western blot procedures and visualization were carried out using standard procedures. The anti-His monoclonal antibody (Thermo Fisher Scientific, Cat. No. MA1-135) and secondary-HRP antibody (Cat. No. 115-035-146; Jackson ImmunoResearch) were diluted in 5% nonfat dried milk in TBS containing 0.1% Tween-20.

### EMSAs

EMSAs were performed as described previously (7), with the modification that 40 fmol of probe and 8 pmol of nonbiotinylated probe competitor were used per reaction. The sequences of all probes used in this study are shown in Table S1. The binding conditions were 75 mM NaCl, 15 mM Tris, pH 7.6, 7.5% glycerol, 2 mM MgCl_2_, 1.5 mM EDTA, 0.1% NP-40, 40 mM DTT, 50 μg BSA, and 1 μg poly(dI-dC) per 20 μl reaction. For most studies, DNA probes were synthesized, biotinylated (when applicable), and purified (either through gel or HPLC) by Thermo Fisher Scientific. Reactions were incubated with the binding buffer and protein on ice for 15 min. Competitor nonbiotinylated probes were then added and the reactions incubated for an additional 20 min at RT. Biotinylated probes were then added, and the reactions incubated for an additional 25 min at RT. The free probes and protein–DNA complexes were separated on 8% polyacrylamide gels and visualized using the LightShift Chemiluminescent EMSA kit (Cat. No. 20148, Thermo Fisher Scientific). Images were collected using Bio-Rad Image Lab software.

For quantitative EMSA studies (*i.e.*, titrations of Alx1-FL, Alx4-FL, and Alx1ΔD2), probes labeled with Cy5 were obtained from Integrated DNA Technologies. The binding conditions were as above, but the amount of Cy5 probe used was 1 nM per reaction. The nanomolar range of protein titration for Alx1-FL and Alx1- ΔD2 was 0, 12, 24, 36, 48, 72, 97, 194, 291, and 388 nM. The nanomolar range for the Alx4-FL was 0, 9, 18, 27, 37, 55, 74, 148, 222, and 296 nM. Gels were scanned using a Typhoon FLA 9000 (GE Healthcare), and bands were quantified using ImageQuant TL from the same company. Background subtractions and band area selections were done according to Altschuler *et al.* (45). The fraction of the probe bound at each protein concentration was calculated as follows: (monomer + dimer)/(monomer + dimer + free probe). The fraction bound as a function of protein concentration was graphed using GraphPad Prism (version 9 for Mac; GraphPad Software; https://graphpad.com). The parameters selected to create the curves were “nonlinear regression” and “two-site–specific binding”.

The active fraction of each protein preparation was determined in a set of binding reactions using a constant concentration of protein (5 nM, as determined above) and increasing concentrations of Cy5-labeled, palindromic oligo, without nonspecific competitor DNA. Bound complexes and free oligo were quantified for each concentration of oligo, and the active concentration of protein was estimated from each pair of data points using the formula shown in Fig. S6. The average of these estimates divided by the initial determination of protein concentration was taken as the active fraction of protein.

For super-shift experiments, we used custom-made, affinity-purified rabbit polyclonal antibodies monospecific for Alx1 and Alx4 (Biomatik). The Alx1 antibody was described previously (7). The Alx4 antibody was raised against the synthetic peptide sequence Cys-TGGVEPIETDRRSHS, and the specificity of the antibody was confirmed by immunoblotting (Fig. S4*B*).

### GFP reporter assays

A 210-bp CRM associated with *Sp-mtmmpb* (ChIP-seq peak 1166) was amplified from *S. purpuratus* genomic DNA and cloned upstream of the basal Sp-Endo16 promoter in the EpGFPII vector (7). Mutations of the putative Alx1-binding sites were generated using the GeneArt Site-Directed Mutagenesis Plus kit (Cat. No. A14604; Thermo Fisher Scientific). Microinjection of reporter constructs was performed, and GFP expression in injected embryos was assayed at the prism stage (48 hours post-fertilization) by fluorescence microscopy, as previously described (7). It is important to note that foreign DNA is incorporated and expressed in a mosaic fashion in sea urchin embryos; this requires the analysis of populations of embryos to reconstruct the expression patterns of transgenes (37). The total number of injected embryos (indicated by the presence of Texas Red dextran), the number of embryos showing PMC-specific GFP expression, the number of embryos showing PMC and ectopic GFP expression, and the number of embryos with only ectopic GFP expression were scored.

## Data availability

All data are in the article and Supporting information or are available upon request from the authors: Jennifer Guerrero-Santoro (jsantoro@andrew.cmu.edu) and Charles A. Ettensohn (ettensohn@cmu.edu) at Carnegie Mellon University.

## Supporting information

This article contains supporting information.

## Conflict of interest

The authors declare that they have no conflicts of interest with the contents of this article.
