# Supplementary material for: Analysis of the DNA-binding properties of Alx1, an evolutionarily conserved regulator of skeletogenesis in echinoderms
Source: J Biol Chem. 2021 Jun 19;297(1):100901. doi: 10.1016/j.jbc.2021.100901 (PMC8319359; doi:10.1016/j.jbc.2021.100901)
Supplement: Supplemental Figures S1–S6 and Table S1 [file mmc1.pdf]

Supporting Information

Analysis of the DNA-binding properties of Alx1, an evolutionarily conserved regulator of skeletogenesis in echinoderms

Jennifer Guerrero-Santoro, Jian Ming Khor, Ayşe Haruka Açıkbaş,
James B. Jaynes, and Charles A. Ettensohn*

Contents:

Table S1 DNA Sequence of the DNA probes used in this study.
Figure S1 Protein samples were separated on SDS-PAGE gels and visualized by Coomassie staining.
Figure S2 The D2 domain of Alx1 is not required for Alx1-Alx1 binding in the absence of DNA.
Figure S3 Alx1 and Alx4 interact in the absence of DNA.
Figure S4 Alx1 and Alx4 from heterodimers on DNA.
Figure S5 EMSA analysis demonstrating the difference between the complex formed on a probe with a palindromic site and a probe containing 2 half sites (Site A and Site B).
Figure S6 Methods for calculating the active fraction of proteins.

Table S1

Probe	Sequence	Used in:
palindromic	5'-Biotin-GTCGGGGCGT**TAAT**AGA**TTTA**AACTTTTTC-3'	Figure 2
palindromic	5'-Cy5-GTCGGGGCGT**TAAT**AGA**TTTA**AACTTTTTC-3'	Figure 3 & 5
palindromic mutant	5'-GTCGGGGCGT**TCGT**AGA**ACGA**AACTTTTTC-3'	Figure 2
Site A	5'-Biotin-GGTATGTATCGTGT**TAAT**CAGCCTGTCTCTT-3'	Figure 2
mutant Site A	5'-GGTATGTATCGTGT**TCGT**CAGCCTGTCTCTT-3'	Figure 2
Site B	5'-Biotin-CTTCATCATCCCGT**TAAT**AAGGACTTGATCG-3'	Figure 2
mutant Site B	5'-CTTCATCATCCCGT**TCGT**AAGGACTTGATCG-3'	Figure 2
Site C	5'-Biotin-ATATCCGCGCGCA**TAAT**GTGACGACGTTAC	Figure 2
mutant Site C	5'-ATATCCGCGCGCA**TCGT**GTGACGACGTTAC-3'	Figure 2
Site D	5'-Biotin-CAAGCTGGTTT**TGAT**TCG**ATCA**CGACTCTCT-3'	Figure 2
mutant Site D	5'-CAAGCTGGTTT**TGCT**TCG**ATCA**CGACTCTCT-3'	Figure 2
longer palindromic (70bp)	5'-Biotin-GTTGATAAACCAACCACAACGTCGGGGCGT**TAAT**AGA**TTTA**AACTTTTTCGTCCGCCTGTAAAGTGATAA-3'	Figure 5 & 6
Site A + Site B (70bp)	5'-Biotin-ATCCAGGTATGTATCGTGT**TAAT**CAGCCTGTCTCTTCATCATCCCGT**TAAT**AAGGACTTGATCGCAAATG-3'	Figure 5 & 6
Site A + Site B (70bp)	5'-Cy5-ATCCAGGTATGTATCGTGT**TAAT**CAGCCTGTCTCTTCATCATCCCGT**TAAT**AAGGACTTGATCGCAAATG-3'	Figure 6
mutant Site A + Site B (70bp)	5'-ATCCAGGTATGTATCGTGT**TCGT**CAGCCTGTCTCTTCATCATCCCGT**TCGT**AAGGACTTGATCGCAAATG-3'	Figure 5 & 6
Site A +++ Site B (70bp)	5'-Biotin-TATGTATCGTGT**TAAT**CAGCCTGTCTCTTCATCATCCCGCTCTTCATCATCCCGT**TAAT**AAGGACTTGAT-3'	Figure 6
Site A +++ Site B (70bp)	5'-Cy5-TATGTATCGTGT**TAAT**CAGCCTGTCTCTTCATCATCCCGCTCTTCATCATCCCGT**TAAT**AAGGACTTGAT-3'	Figure 6
mutant Site A +++ Site B(70bp)	5'-TATGTATCGTGT**TCGT**CAGCCTGTCTCTTCATCATCCCGCTCTTCATCATCCCGT**TCGT**AAGGACTTGAT-3'	Figure 6
shorter palindromic (55bp)	5'-Biotin-ACCAACCACAACGTCGGGGCGT**TAAT**AGA**TTTA**AACTTTTTCGTCCGCCTG-3'	Figure S5
shorter palindromic mutant (55bp)	5'-Biotin-ACCAACCACAACGTCGGGGCGT**TCGT**AGA**ACGA**AACTTTTTCGTCCGCCTG-3'	Figure S5
shorter Site A + Site B (55bp)	5'-Biotin-TATGTATCGTGT**TAAT**CAGCCTGTCTCTTCATCATCCCGT**TAAT**AAGGACT-3	Figure S5
shorter mutant Site A +Site B (55bp)	5'-TATGTATCGTGT**TCGT**CAGCCTGTCTCTTCATCATCCCGT**TCGT**AAGGACTTGAT-3	Figure S5

Figure S1

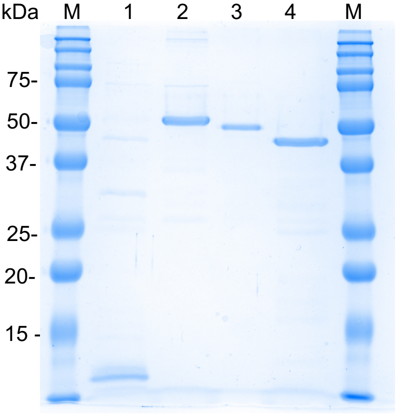

Figure S1- The protein samples were separated on SDS-PAGE gels and visualized by Coomassie staining.
Lane M- marker, lane 1- Alx1-HD, lane 2-Alx1-FL, lane 3-Alx1-ΔD2 and lane 4-Alx4-FL and lane M-marker.

Figure S2

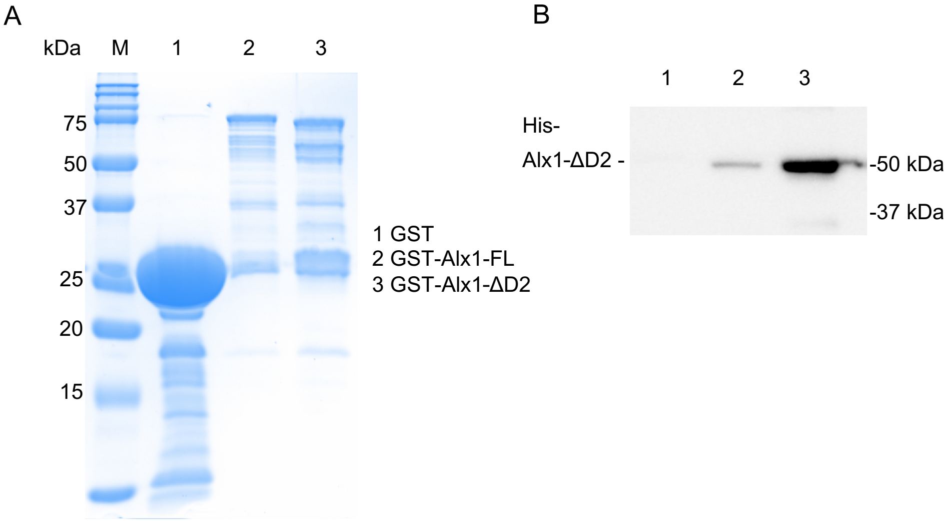

Figure S2- The D2 domain of Alx1 is not required for Alx1-Alx1 binding in the absence of DNA. A) GST, GST-tagged Alx1-FL protein and GST-Alx1-ΔD2 were expressed in *E. coli* and immobilized on glutathione beads (Lanes 1-3 respectively). The protein samples were separated on SDS-PAGE gels and visualized by Coomassie staining. B) Immobilized proteins from A were incubated with purified His-Alx1-ΔD2. The beads were washed, separated on an SDS-PAGE gel, and analyzed by Western blotting using an anti-His antibody.

Figure S3

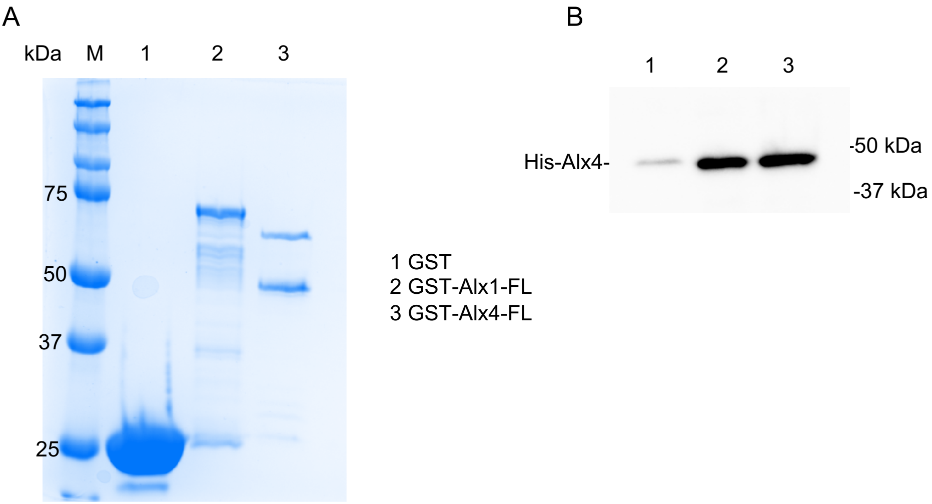

Figure S3 - Alx1 and Alx4 interact in the absence of DNA. A) GST, GST-tagged Alx1 protein and GST-Alx4 were expressed in *E. coli* and immobilized on glutathione beads (Lanes 1-3 respectively). The protein samples were separated on SDS-PAGE gels and visualized by Coomassie staining. B) Immobilized proteins from A were incubated with purified His-Alx4. The beads were washed, separated on an SDS-PAGE gel, and analyzed by Western blotting using an anti-His antibody.

Figure S4

A

Lane	1	2	3	4	5	6	7
Alx1-FL (nM)	24	-	-	24	24	24	24
Alx4-FL (nM)	-	19	149	19	74	149	223

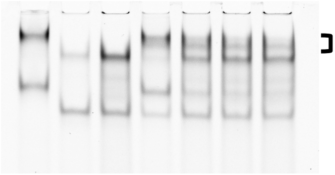

B

Lane	1	2	3
Alx1-FL (ng)	49	-	-
Alx4-FL (ng)	-	37	9

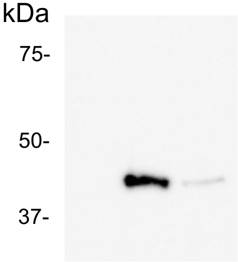

C

Lane	1	2	3	4	5	6	7	8	9	10
Alx1-FL (nM)	24	-	24	24	24	24	24	24	24	24
Alx4-FL (nM)	-	74	74	74	74	-	149	149	149	149
Alx1 antibody (μg)	-	-	-	0.5	-	-	-	-	0.5	-
Alx4 antibody (μg)	-	-	-	-	0.5	-	-	-	-	0.5

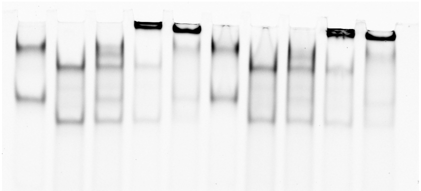

Figure S4- EMSA analysis showing the formation of heterodimers on the *Sp-EMI/TM* palindromic site. A) Formation of homodimers for Alx1-FL and Alx4-FL (lanes 1-3). Formation of heterodimers of Alx1-FL and Alx4-FL (lanes 4-7). Bracket to the right of Panel A marks the heterodimer signal.
B) Validation of the antibody specific for Alx4-FL. Western Blot of purified proteins Alx1-FL and Alx4-FL. C) Supershift of Alx1-FL and Alx4-FL by antibodies against the same proteins (lanes 4,5,9 and 10).

Figure S5

Palindromic site
CAACGTCGGGGCGT**TAAT**AGA**TTTA**AACTTTTTCGTCCG
Site A + Site B
GTGT**TAAT**CAGCCTGTCTCTTCATCATCCCGT**TAAT**AAG

Lane	1	2	3	4	5	6
Palindromic probe (55bp)	+	+	+	-	-	-
Site A + Site B probe (55bp)	-	-	-	+	+	+
WT competitor	-	200X	-	-	200X	-
Mutant competitor	-	-	200X	-	-	200X

F>

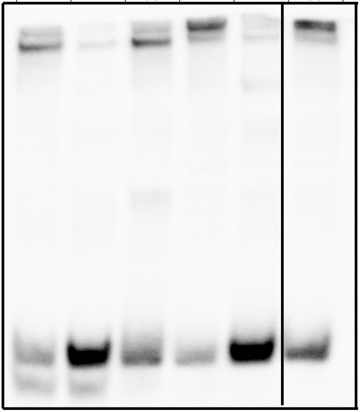

Figure S5- EMSA analysis demonstrating the difference between the complex formed on a probe with a palindromic site and a probe containing two half sites (Site A and Site B). Binding specificity was confirmed by adding a wild type or mutant competitor. Vertical line within the gel image indicates spliced-out lanes. For the complete sequence of all probes used in this study, see Table S1. F = free probe.

Figure S6

The following is a description of how to get the total [active DNA binding protein] (p) from two data points, without knowing or determining the dissociation constant (k).

The data points come from measuring the [dimer] and [monomer] bound complexes and the free [substrate oligo], for a constant amount of added protein, and different amounts of added oligo.

The substrate oligo is assumed to have two equivalent specific binding sites for the protein. k = dissociation constant for each single protein complex (protein bound to probe DNA at either site);

f = [free protein],
p = [total protein],
a = [single site-bound complex], (there are two such complexes, but they are represented by a single variable, because they are indistinguishable),
A = [double site-bound complex].

$$f = \frac{ka}{2h}$$

since

$$k = \frac{2fh}{a}$$

so

$$f + a = \left(\frac{k}{2h} + 1\right) a = p - 2A$$

since

$$p = f + a + 2A$$

Method 1:
Take two data points, (a, A, h) and a 2nd data point, designated (b, B, j).

Use the 2nd of these to eliminate k from the above equation, then solve for p:

$$\left(\frac{k}{2h} + 1\right) a = p - 2A$$

$$2\left(\frac{p - 2A}{a} - 1\right) h = k$$

$$2\left(\frac{p - 2B}{b} - 1\right) j = k = 2\left(\frac{p - 2A}{a} - 1\right) h$$

$$\frac{p}{b} j - \left(\frac{2B}{b} + 1\right) j = \frac{p}{a} h - \left(\frac{2A}{a} + 1\right) h$$

$$p\left(\frac{j}{b} - \frac{h}{a}\right) = \left(\frac{2B}{b} + 1\right) j - \left(\frac{2A}{a} + 1\right) h$$

$$p = \frac{\left(\frac{2B}{b} + 1\right) j - \left(\frac{2A}{a} + 1\right) h}{\frac{j}{b} - \frac{h}{a}}$$

This can be done pairwise for the entire set of data points, to give a set of semi-independent determinations of p.

The variation among this set of determinations of p can be used as an indication of the error associated with the determined value.

Method 2:

To get p as a parameter in graphing (curve fitting), as a function of a, A, and h, take one of the data points as a given (the "anchor point" below) in order to eliminate k, and then input the others as sets of variables, with p as a parameter to be determined by curve fitting:

$$2\left(\frac{p-2A}{a}-1\right)h = k$$

As above, the "anchor" data point is (b, B, j):

$$2\left(\frac{p-2B}{b}-1\right)j = k$$

Substitute this into

$$\left(\frac{k}{2h}+1\right)a = p-2A$$

$$a = \frac{p-2A}{\frac{k}{2h}+1}$$

$$a = \frac{p-2A}{\dfrac{2\left(\dfrac{p-2B}{b}-1\right)j}{2h}+1} = \frac{p-2A}{\dfrac{\left(\dfrac{p-2B}{b}-1\right)j}{h}+1}$$

Let the dependent variable y = a, and let independent variables (x1, x2) = (A, h).

Feed these along with the above equation, in which (b, B, j) is taken from one of the data points, into non-linear curve fitting software.

Alternatively, use the equation

$$\left(\frac{k}{2h}+1\right)a = p-2A$$

$$\frac{k}{2h} = \frac{p-2A}{a}-1 = \frac{2\left(\dfrac{p-2B}{b}-1\right)j}{2h} = \left(\frac{p-2B}{b}-1\right)\frac{j}{h}$$

$$h = j\frac{\dfrac{p-2B}{b}-1}{\dfrac{p-2A}{a}-1}$$

where y = h, independent variables are (x1, x2) = (a, A), known quantities are b, B, and j, and p is the parameter to be found by non-linear curve fitting (e.g., at https://statpages.info/nonlin.html).

A separate determination of p can be obtained using each data point as the anchor, and the other data points as the set of independent and dependent variables.

As for Method 1, the variation among this set of determinations of p can be used as an indication of the error associated with the determined value.

After using the above equation

$$k = 2\left(\frac{p - 2A}{a} - 1\right) h$$

to get k after p has been determined, one can check the goodness of fit by doing a 2-parameter curve fit to find p and k using the data points and the equation

$$h = \frac{k}{2\left(\frac{p - 2A}{a} - 1\right)}$$

The initial guesses for the parameters can be the values of p and k determined above.

Assuming the curve fitting converges to values close to those determined by Method 1 or Method 2, an independent estimate of the error associated with the determination of p can be obtained from this procedure.
